# Editorial: Engineering Microbes for Therapy

**DOI:** 10.3389/fmicb.2019.00629

**Published:** 2019-04-02

**Authors:** Aleš Berlec, Borut Štrukelj

**Affiliations:** ^1^Department of Biotechnology, Jožef Stefan Institute, Ljubljana, Slovenia; ^2^Faculty of Pharmacy, University of Ljubljana, Ljubljana, Slovenia

**Keywords:** probiotics, therapy, engineering, *Lactococcus*, *Lactobacillus*, vaccination, immunotherapy, delivery

The notion that microbes are not just vicious pathogens but can play protective role in human health has been well-established in recent years. The concept of probiotics, “live micro-organisms which, when administered in adequate amounts, confer a health benefit on the host,” has been introduced, focusing mainly on species of lactic acid bacteria already present in food products. The beneficial activity of selected probiotics has been well-documented and supported in clinical trials. Probiotics have an important economic impact in food, food supplement, and veterinary industry with increasing market size.

The introduction of next generation sequencing techniques has boosted the research of human microbiota and established the importance of its composition. The perturbation of composition of microbiota can lead to imbalance and dysbiosis—a disease characterized by the changes in microbiota. The latter has been demonstrated in inflammatory bowel disease, irritable bowel syndrome, allergy, obesity, colon cancer, and even autism. The ability to restore the balance with microbial intervention was also demonstrated. The stunning example is fecal transplant for the treatment of recurrent *Clostridium difficile* infection.

Engineering microbes for therapy can lead to selection of new microbial strains and mixtures, or targeted improvement of existing microbial strains, achieved by mutagenesis, genetic engineering, and synthetic biology. The new species will exceed the existent probiotic definition for food applications, but may well be adopted by the pharmaceutical industry as “pharmabiotics” or “live biotherapeutic products.”

Delivery of antigens for the purpose of vaccination is maybe among the most frequently explored ways of using genetically engineered microbes, although not many examples have actually reached the human clinical stage. Prosperi de Castro et al. have reviewed the use of lactic acid bacterium *Lactococcus lactis* as a mucosal vaccine delivery vehicle. Special emphasis was put on the delivery of DNA vaccines that enable *in situ* translation of the antigen. Novel, advanced and food-grade vectors have been developed for that purpose. Apart from *L. lactis*, Ramos-Vega et al. have reviewed the use of another, much less established microbial host—microalgae from the genus *Schizochytrium*. These were shown as favorable hosts for influenza subunit vaccine production, and were described as safe for human consumption. Apart from delivery of antigens, the microbes can also be engineered to deliver allergens with the final goal of desensitizing allergic individuals, essentially enabling immunotherapy of allergic disease. This field has been thoroughly described by Zahirović and Lunder.

Other possible uses of engineered microbes include immunomodulation, inflammation, cancer, infectious diseases, and metabolic disorders. Some recent examples from this field have been reviewed by Bron and Kleerebezem. Jacouton et al. have developed *L. lactis* secreting IL-17A that prevented the formation of tumors in a mouse model. Mauras et al. have developed new plasmid vector for *Bifidobacterium bifidum* and used it to express Interleukin-10. These recombinant bacteria were effective in decreasing intestinal inflammation in mice. Therapeutic proteins, delivered by engineered microbes, can be either secreted or displayed on the surface. Some species from the genus *Lactobacillus* contain surface (S) layers that are made of crystalline arrays of repeating subunits of S layer proteins. Klotz and Barrangou reviewed the possibilities to apply these structures for biotherapeutic applications. Apart from engineering microbes to deliver therapeutic proteins, they can also be used to activate prodrugs at a defined site of action, by delivering appropriate enzymes, thereby minimizing side effects. Aučynaitė et al. report the discovery of deaminases that convert 5-Fluoroisocytosine into 5-Fluorouracil.

Engineering of microbes can also encompass the development and improvement of their dosage forms. Cordeiro et al. have improved the viability of *Lactobacillus casei* BL23 and *Propionibacterium freudenreichii* 138 in fermented skim milk by the addition of whey protein isolate. The latter has increased the protective activity of the beverage against mucositis in mice. Another mode of protection of bacteria, namely incorporation of *L. lactis* in sodium alginate microcapsules, has been reported by Coelho-Rocha et al. Considerably higher expression of the model protein in mice gastrointestinal tract has been achieved.

The studies of engineering of microbes do not focus only on expressing heterologous proteins, but also on the role of intrinsic bacterial proteins. *Propionibacterium freudenreichii* is a beneficial Gram-positive bacterium, currently considered as an emerging probiotic with promising immunomodulatory properties. do Carmo et al. have shown that its S layer protein SlpB has a crucial pleiotropic role in mediating beneficial activity. Inactivating SlpB led to dramatic change of bacterial surface properties, as well as on the entire bacterial proteome. Aucouturier et al. have shown the importance of prophages in the genome of *Lactococcus lactis* ssp. *lactis* IL1403. Their removal contributed significantly to the changes in the lactococcal physiology, and may play a role in the adaptation of bacteria to the changes in their environment.

The use of engineered microbes is not limited to human medicine only, but also includes veterinary applications. Hu et al. reported a standardized preparation for fecal transplantation of microbiota in pigs. Such protocols are crucial for establishing stool banks of appropriate safety, and thereby promoting this approach as an alternative to antibiotics for intestinal health of animals.

The compendium of original and review articles that were assembled in the Research Topic Engineering microbes for therapy represents an up to date overview, shows new results, as well as demonstrates future trends. These and other studies suggest that the therapeutic use of microbes has exciting future. However, the crucial step from successful proof-of-principle reports in animal models to well-executed human clinical trials is, at the moment, still lacking in most of the cases and represents a challenge for the future.

## Author Contributions

All authors listed have made a substantial, direct and intellectual contribution to the work, and approved it for publication.

### Conflict of Interest Statement

The authors declare that the research was conducted in the absence of any commercial or financial relationships that could be construed as a potential conflict of interest.

